# Stability of bacteriophages in burn wound care products

**DOI:** 10.1371/journal.pone.0182121

**Published:** 2017-07-27

**Authors:** Maia Merabishvili, Riet Monserez, Jonas van Belleghem, Thomas Rose, Serge Jennes, Daniel De Vos, Gilbert Verbeken, Mario Vaneechoutte, Jean-Paul Pirnay

**Affiliations:** 1 Laboratory for Molecular and Cellular Technology, Queen Astrid Military Hospital, Brussels, Belgium; 2 Laboratory for Bacteriology Research, Faculty Medicine & Health Sciences, Ghent University, Ghent, Belgium; 3 George Eliava Institute of Bacteriophages, Microbiology and Virology, Tbilisi, Georgia; 4 Hospital Pharmacy, Queen Astrid Military Hospital, Brussels, Belgium; 5 Burn Wound Center, Queen Astrid Military Hospital, Brussels, Belgium; Institute of Immunology and Experimental Therapy, Polish Academy of Sciences, POLAND

## Abstract

Bacteriophages could be used along with burn wound care products to enhance antimicrobial pressure during treatment. However, some of the components of the topical antimicrobials that are traditionally used for the prevention and treatment of burn wound infection might affect the activity of phages. Therefore, it is imperative to determine the counteraction of therapeutic phage preparations by burn wound care products before application in patients. Five phages, representatives of two morphological families (*Myoviridae* and *Podoviridae)* and active against 3 common bacterial burn wound pathogens (*Acinetobacter baumannii*, *Pseudomonas aeruginosa* and *Staphylococcus aureus*) were tested against 13 different products commonly used in the treatment of burn wounds. The inactivation of the phages was quite variable for different phages and different products. Majority of the anti-infective products affected phage activity negatively either immediately or in the course of time, although impact was not always significant. Products with high acidity had the most adverse effect on phages. Our findings demonstrate that during combined treatment the choice of phages and wound care products must be carefully defined in advance.

## Introduction

Almost all victims of burns have a more or less severe damage to the skin. The skin is our largest organ and is vital in the host’s protection against infection. In addition, the depression of immune responses induced by severe burns predisposes burn patients to infectious complications. Immediately after the accident, the burn will usually show little or no growth of microorganisms as a consequence of the thermal insult, but in the first days after the burn, wounds usually become colonized with gram-positive bacteria such as commensal skin staphylococci, which are located deep within sweat glands and hair follicles and survived the thermal injury. After an average of 5–7 days, gram-negative bacteria and yeasts, mostly from the host's normal gastrointestinal and upper respiratory microflora and/or from the hospital environment, also colonize the burn wounds [1].

The introduction of penicillin G in the 1950s resulted in the introduction of *Staphylococcus aureus* as the major cause of infection in burn wound patients. Today, *S*. *aureus* remains a common cause of early burn wound infection, but *Pseudomonas aeruginosa* from the patient’s gastrointestinal tract and/or the hospital environment has become the most common burn wound pathogen [2]. The incidence of infections due to other inherently antimicrobial resistant gram-negative bacteria, such as *Acinetobacter baumannii*, *Enterobacter* spp. and *Klebsiella pneumoniae* and of fungi has also increased in past decades. Due to their capacity for long-term survival in the hospital environment, combined with a formidable inherent and acquired antibiotic resistance, some *A*. *baumannii* strains have emerged as important nosocomial pathogens, especially in critically ill patients, as found in intensive care units and burn wound centers [3]. Multidrug resistant strains of *A*. *baumannii*, referred to as ‘Iraqibacter’ due to their emergence in military treatment facilities during the Iraq War, have spread to civilian hospitals due to the transfer of infected soldiers [4].

Colonization and infection of the burn wound by microorganisms can have disastrous consequences. The presence of bacteria such as *P*. *aeruginosa* can seriously hamper skin graft take [5] and, even though modern medical care has significantly reduced the mortality among thermally injured patients, most deaths in severely burn-injured patients are due to burn wound sepsis [1]. Different topical burn wound care products and techniques, such as silver and iodine-containing creams, whether or not in combination with systemic antibiotics, have been developed to prevent colonization and infection. However, these treatment methods are not always effective, not least due to the increasing antimicrobial resistance in, amongst others, burn wound pathogens. The introduction of new drugs and treatments against antimicrobial resistant infections should be accelerated. One of the ‘new’ treatments that is increasingly considered is phage therapy [6–8], i.e. the use of bacterial viruses named bacteriophages (phages for short) to treat bacterial infection. In recent years we designed phage cocktails for the treatment of *A*. *baumannii*, *P*. *aeruginosa* and *S*. *aureus* infections in burn wound patients [9,10]. The most important limitation of phages is that they are host- or even strain-specific. Clinicians must first know which bacteria (or bacterial strains) are causing the infection before treating the patient with phages. In addition, it was shown that burn wounds are often simultaneously colonized with different bacterial species (polymicrobial) and/or strains (polyclonal) [11]. To prevent that a number of burn wound colonizing or infecting bacterial species or strains would not be targeted, phage therapy would best be combined with conventional topical burn wound care products and where warranted with systemic antibiotics. In this context, the therapeutic potential of phages for topical burn wound treatment will partly be determined by their stability in burn wound care products. In the present study we determined the stability of five therapeutically interesting phages against *A*. *baumannii* (Acibel004 and Acibel007), *P*. *aeruginosa* (PNM and 14–1) and *S*. *aureus* (ISP) [9,10,12] in 13 common burn wound care products (Bactroban, colistin milk, Flammazine, Flaminal Hydro and Flaminal Forte, Fucidin, Furacin, Hibidil, Intrasite Gel, iso-Betadine Gel, P.O.H. (polymyxin-oxytetracyclene-hydrocortisone), Sulfamylon). These stability data are required for phages to be used within a burn wound setting.

## Materials and methods

### Bacteriophages and bacterial strains

Five bacteriophages, i.e. *A*. *baumannii* phages Acibel004 and Acibel007, *P*. *aeruginosa* phages PNM and 14–1 and *S*. *aureus* phage ISP were tested for their stability in different wound care products. The phages ISP, PNM and 14–1 are components of a therapeutic bacteriophage cocktail BFC1, previously developed [9]. The phages Acibel004 and Acibel007, active against *A*. *baumannii*, were previously isolated and characterized by our group [10]. All bacteriophages were propagated by the agar-overlay method [9] to the titer of 11 log pfu/ml and then diluted in non-buffered physiological saline (0.9% NaCl) to the final titer of 9 log pfu/ml. Luria Bertani (LB) broth and agar media (Becton Dickinson, Erembodegem, Belgium) were used for propagation and culturing bacteria and phages. Characteristics of bacteriophages used in the study are presented in Table 1.

**Table 1:** Characteristics of bacteriophages used in the stability study.

Phage Name	ISP	PNM	14–1	Acibel004	Acibel007
**Host species**	*S*. *aureus*	*P*. *aeruginosa*	*P*. *aeruginosa*	*A*. *baumannii*	*A*. *baumannii*
**Source of isolation**	Unknown	River water	Sewage water	Sewage water	Sewage water
**Place of isolation**	Tbilisi, Georgia	Tbilisi, Georgia	Regensburg, Germany	Ghent, Belgium	Ghent, Belgium
**Date of isolation**	1920–30	1999	2000	2010	2010
**Family/genus of Caudovirales**	Myoviridae *Twortvirus*	Podoviridae *Phikmvvirus*	Myoviridae *Pbunavirus*	Myoviridae ND	Podoviridae *Phikmvvirus*
**Genome size (kbp)**	138.3	42.4	66.2	99.7	42.7
**Accession No.**	FR852584	NA	NC_011703	KJ473422	KJ473423

NA, non-applicable; ND, not defined.

Three bacterial strains were used in the study as host strains for propagation of the relevant bacteriophages: *A*. *baumannii* 070517/0072 [10], *P*. *aeruginosa* 573 [9] and *S*. *aureus* ATCC 6538.

### Burn wound care products and their active ingredients

In total, 13 different topical wound care products were used in the study. Detailed information on the applied products is presented in Table 2. Bactroban, colistin milk, P.O.H. and Sulfamylon cream were produced by the pharmacy department of the Queen Astrid Military Hospital (QAMH). Flaminal Forte and Flaminal Hydro (Flen Pharma, Kontich, Belgium), Flammazine 1% (Sinclair Pharmaceuticals, Godalming, UK), Fucidin (Leo Pharma, Lier, Belgium), Furacin (Limacom nv-Pharma Division, Diepenbeek, Belgium), Hibidil (Regent Medical, Irlam, UK), Intrasite Gel and Iruxol (TJ Smith & Nephew, Hull, UK), iso-Betadine Gel 10% (Meda Pharma, Brussels, Belgium) were obtained from the pharmacy of the QAMH.

**Table 2:** Characteristics of burn wound products used in the stability study presented in alphabetical order.

Wound Care Products	Producer	Type of preparation	Composition	Indication	Active Ingredients	Mechanism of activity of active ingredient	Target Groups for anti-infective agents	Activity mode of the active anti-infective agents
**Bactroban**	MHPh	Ointment	Mupirocin 2%, PEG 400, PEG 3350	Anti-infective	Mupirocin	Mupirocin blocks the integration of isoleucine into the peptide during synthesis via mimicking the epoxy moiety of monic acid and isolecine tRNA synthetase.	Gram-positive and gram-negative bacteria, mycoplasmas	Bacteriostatic
**Colistin milk**	MHPh	Suspension	Polymyxin E 0.5%, paraffin, Tween 80, cetyl alcohol, labrafyl, lactic acid, water	Anti-infective	Polymyxin E	Polymyxin E to LPS and phospholipids in the outer cell membrane of gram-negative bacteria displacing divalent cations (Ca^2+^ and Mg^2+^) from the phosphate groups of membrane lipids leading to disruption of the outer cell membrane and leakage of intracellular contents.	Gram-negative bacteria	Bactericidal
**Flaminal Forte**	Flen Pharma	Gel	Alginate 5.5%, PEG, GLG enzyme system, potassium sorbate, potassium iodide, buffer, water	Wound debriding and anti-infective	Alginate	Alginates absorb and lock in exudate and retains a humid environment.	Gram-positive and gram-negative bacteria	Bactericidal
					**GLG enzyme system**	The GLG enzyme system disrupts bacterial cells via oxidation of cell wall components.		
**Flaminal Hydro**	Flen Pharma	Gel	Alginate 3.5%, PEG, hydroxypropylcellulose, GLG enzyme system, potassium sorbate, potassium iodide, buffer, water	Wound debriding and anti-infective	Alginate	Alginates absorb and lock in exudate and retain a humid environment.	Gram-positive and gram-negative bacteria	Bactericidal
					**GLG enzyme system**	The GLG enzyme system disrupts bacterial cells via oxidation of cell wall components.		
**Flammazine**	Sinclair Pharmaceuticals Ltd	Cream	Silver sulfadiazine 1%, polysorbaat 60, polysorbaat 80, glycerylmonostearaat, cetyl alcohol, liquid paraffin, propylenglycol, water	Anti-infective	Silver sulfadiazine	Silver ions bind to nucleophilic amino acids, sulfhydryl, amino, imidazole, phosphate, and carboxyl groups in proteins, causing protein denaturation and enzyme inhibition while released sulfadiazine inhibits folic acid synthesis in bacteria.	Gram-positive and gram-negative bacteria, fungi	Bactericidal
**Fucidin**	Leo Pharma	Cream	Fusidic acid 2%, butyhydroxyanisol, cetylalcohol, glycerol, liquid paraffin, kalium sorbaat, polysorbaat 60, white vaseline, all-rac-alpha-tocopherol, hydrochloric acid, water	Anti-infective and anti-inflammatory	Fusidic acid	Fusidic acid inhibits protein synthesis at the translation stage targeting elongation factor G.	Gram-positive and gram-negative bacteria, parasites	Bactericidal or -static, depeding on concentration
					**Hydrocortisone**	Hydrocortisone binds to the glucocorticoid receptor (GR). The activated GR complex, in turn, up-regulates the expression of anti-inflammatory proteins in the nucleus and represses the expression of proinflammatory proteins in the cytosol by preventing the translocation of other transcription factors from the cytosol into the nucleus.		
**Furacin**	Limacom -Pharma division	Ointment	Nitrofurazone 0.2%, PEG 300, PEG 1000, PEG 3000	Anti-infective	Nitrofurazone	Nitofurazone inhibits aerobic and anaerobic metabolism and lowers the ATP levels in bacteria.	Gram-positive and gram-negative bacteria, trypanosoma; fungi	Bactericidal or -static, depeding on concentration
**Hibidil**	Regent medical	Solution	Chlorhexidine gluconate 0.05%, Nonoxinol 9, ethanol, Azorubine, sodium hydroxyde, D-gluconolactone, water	Anti-infective	Chlorhexidine	Chlorhexidine adheres to the microorganism’s cell wall and disrupts the integrity of the cell membrane causing the leakage of intracellular components of the organisms.	Gram-positive and gram-negative bacteria	Bactericidal
**Intrasite Gel**	TJ Smith & Nephew	Gel	Carboxymethylcellulose polymer 2.3%, propylene glycol 20%, water	Wound debriding	Carboxymethylcellulose	Carboxymethylcellulose absorbs and locks in exudate and retains a humid environment.	NA	NA
**Iruxol**	TJ Smith & Nephew	Ointment	Collagenase Knoll (1.0–4.75 mg) with at least 1.2 U clostridiopeptidase A and at least 0.24 U other proteases, liquid paraffin, soft white paraffin	Wound debriding	Collagenase Knoll	Collagenase Knoll digests all protein components of the wound, ensuring chemical debridement.	NA	NA
**iso-Betadine Gel**	Meda Pharma	Gel	Povidone-iodine 10%, PEG 400, PEG 4000, PEG 6000, water	Anti-infective	Povidone-iodine	Povidone-iodine kills prokaryotic and eukaryotic cells through iodination of lipids and oxidation of cytoplasmic and membrane compounds.	Viruses, bacteria, protozoa, fungi	Microbicidal
**P.O.H.**	MHPh	Ointment	Oxytetracycline hydrochloridum 0.5%, polymyxin B 0.12%, hydrocortisone 1%, liquid paraffin, white vaseline	Anti-infective and anti-inflammatory	Oxytetracycline	Oxytetracycline inhibits protein synthesis via binding to 30S ribosomal subunit and interrupting tRNA and mRNA interaction.	Gram-positive and gram-negative bacteria, mycoplasmas, chlamydiae, spirochetes	Bacteriostatic
					**Polymyxin B**	Polymyxin B binds to LPS and phospholipids in the outer cell membrane of gram-negative bacteria displacing divalent cations (Ca^2+^ and Mg^2+^) from the phosphate groups of membrane lipids leading to disruption of the outer cell membrane and leakage of intracellular contents.	Gram-negative bacteria	Bactericidal
					**Hydrocortisone**	Hydrocortisone binds to the glucocorticoid receptor (GR). The activated GR complex, in turn, up-regulates the expression of anti-inflammatory proteins in the nucleus and represses the expression of proinflammatory proteins in the cytosol by preventing the translocation of other transcription factors from the cytosol into the nucleus.	NA	NA
**Sulfamylon**	MHPh	Cream	Mafenide acetate 8.5%, cetyl alcohol, stearyl alcohol, polyoxyl 40 stearate, PEG-8 stearate, glycerin, methylparaben, propylparaben, sodium metabisulfite, EDTA, water	Antibacterial	Mafenide acetate	The exact mechanism of action is unknown.	Gram-positive and gram-negative bacteria	Bacteriostatic

ATP, adenosine triphosphate; EDTA, Ethylenediaminetetraacetic acid; U, Enzyme Unit; GLG, Glucose oxidase—Lactoperoxidase—Guaiacol; GR, glucocorticoid receptor; LPS, lipopolysacharides; MHPh, military hospital pharmacy; PEG, polyethylene glycol; RNA, ribonucleic acid

NA, non-applicable.

Five active ingredients of four wound care products (Bactroban, Hibidil, iso-Betadine Gel and P.O.H.) were also tested (Table 2): mupirocin (Alfa Aesar, Massachusetts, U.S.), chlorhexidine gluconate (MP Biomedicals, Illkrich, France), povidone-iodine (Acros Organics, New Jersey, USA), oxytetracycline hydrochloridum (Sigma-Aldrich, Steinheim, Germany) and polymyxin B (Fagron NV, Waregem, Belgium).

### Stability assays

Products were mixed with each phage suspension (9.0 log pfu/ml) at a ratio of 1:1, volume per volume (v/v) for products of liquid consistency (colistin milk, Hibidil and solutions of the five selected active ingredients) or weight per volume (w/v) for the creams and ointments. The five active ingredients were diluted in physiological saline (NaCl 0.9%) to the concentration at which they are present in the wound care products.

Mixtures were further incubated at 37°C for 2, 4 and 24 h, where after the phages were quantified by the agar-overlay method [9]. As a control, prior to the stability experiments each phage was checked for its stability in physiological solution at 37°C for 24 h. Effective therapeutic titer (ETT) was defined as 7.0 log pfu/ml. For Bactroban ointment, colistin milk, Hibidil solution, iso-Betadine Gel, P.O.H., mupirocin, oxytetracycline hydrochloridum and povidone-iodine the detection threshold (DT) of phage activity had to be defined as the lowest dilution of the product or active ingredient, at which bacterial strains still form confluent growth loan on Petri dishes with LB agar. Below the DT, the growth of host bacteria is too much affected by the antimicrobial activity of the product to allow an adequate assessment of phage activity. Each test was performed in triplicate and mean values were calculated.

### Measurement of pH

For 7 products (Flaminal Forte, Flaminal Hydro, Flammazine, Fucidin, Furacin, Iruxol, iso-Betadine Gel) pH values were taken from the certificates of analysis. For the remaining products and the 5 selected active ingredients, pH values were tested by pH-meter HACH series HQ40d with INTELLICAL^TM^ PHC101 electrode at 26°C in triplicate, mean values and standard deviations were calculated.

## Results and discussion

We previously developed a well-defined phage cocktail active against *P*. *aeruginosa* and *S*. *aureus* [9] and applied it in a small pilot clinical trial in burn wound patients [13]. Several hurdles and pitfalls were encountered during this study, which hampered an adequate evaluation of the efficacy of the phage cocktail. Nevertheless, the perspectives of phage therapy in burn wound treatment are regarded as promising, especially in the light of the increasing number of drug-resistant infections in this type of patients [1,14].

Because burn wounds are most often colonized or even infected by multiple bacterial species or strains, it is likely that most of the future phage therapy and clinical trial protocols will provide for the simultaneous use of phage preparations and conventional topical antimicrobials. Moreover, current conventional burn wound care protocols imply the application of different agents that ensure hydration and debridement in addition to an antimicrobial activity.

Phages will probably always be used in conjunction with several other medical approaches in burn wound care, and therefore, it is important to determine the stability of phages in the presence of other care products.

A broad variety of creams and ointments containing natural and synthetic antibiotics or anti-infective agents are available. Hydration and subsequent debridement of the wounds are often provided by hydrogels. A high variety of hydrogels is available on the pharmaceutical market and some of them (e.g. Flaminal Hydro) also exhibit antimicrobial properties. Some antimicrobial topicals may also contain also corticosteroids conferring an anti-inflammatory effect.

In the present study we included a large variety of burn wound care products, including hydrogels.

Because it is generally accepted that therapeutic phage preparations should contain active phage particles in concentrations of 6.0–7.0 log pfu/ml [15], we considered 7.0 log pfu/ml as an effective therapeutic titre (ETT). ETT is recommended to be present in therapeutic phage cocktails after incubation with the wound care products.

Four topical wound care products used in this study, i.e. Bactroban ointment, colistin milk, Fucidin cream and P.O.H. ointment, contain antibiotics produced by different microorganisms as the main active component along with other additives, including steroids. Each of these products had variable activity on the tested phages.

Bactroban cream contains 2% mupirocin, a bacteriostatic antibiotic of the monoxycarbolic acid class, produced by *Pseudomonas fluorescens*. The mechanism of its activity implies blocking the integration of isoleucine into the peptide during synthesis via mimicking the epoxy moiety of monic acid and isoleucine tRNA synthetase [16–19]. Mupirocin has good activity against gram-positive cocci and mycoplasmas. Although mupirocin lacks the ability to penetrate the cell wall of gram-negative bacilli, it is still active against some gram-negative cocci and coccobacilli, such as *Bordetella pertussis*, *Haemophilus influenzae*, *Moraxella catarrhalis*, and *Pasteurella multocida* [19].

**Fig 1:**
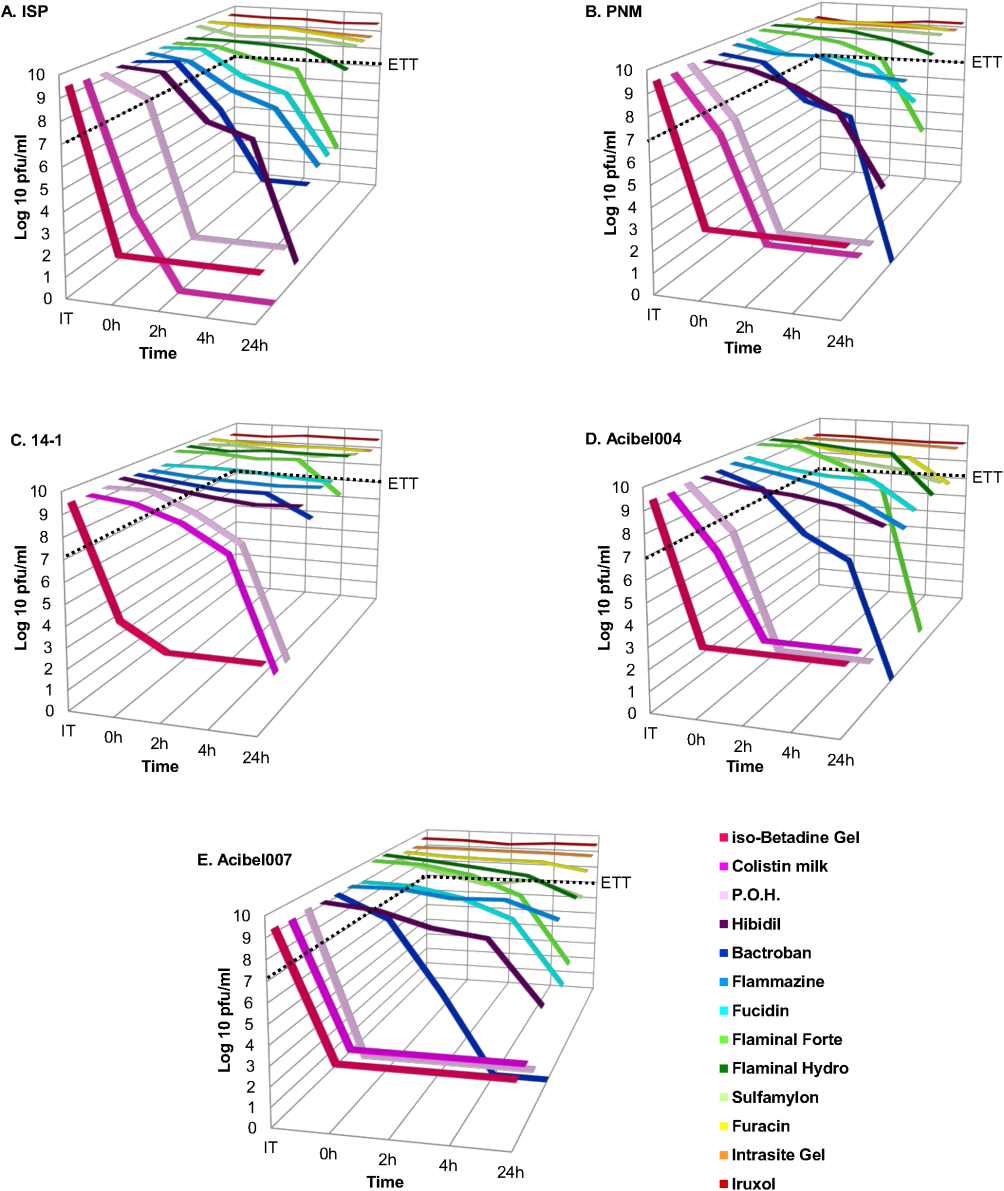
Stability of bacteriophages in burn wound care products. (A) ISP phage; (B) PNM phage; (C) 14–1 Phage; (D) Acibel004 phage; (E) Acibel007 phage. The results are the mean values of three titrations. ETT: effective therapeutic titer (7.0 log pfu/ml). The wound care products are depicted in declining order of their inhibition activity.

Bactroban affected the activity of phages already after 2 hours of incubation, causing a gradual decrease in activity with complete inactivation (or activity below the DT) for all phages after 4 hours, except for myovirus 14–1, which maintained activity above the ETT even after 24 hours of incubation (Fig 1, S1 Fig). Bactroban’s active ingredient mupirocin, tested separately at the concentration of 2%, showed a higher inactivation degree than the final product. All phages, except 14–1, were inactivated below the ETT already after 2 hours of incubation. Only phage 14–1 maintained its activity above the ETT even after 24 hours, similar to Bactroban. The higher inactivation degree of mupirocin could be due to its much lower pH value (3.8) in comparison to Bactroban (5.9) (Table 3).

**Table 3:** pH values of wound care products and their active ingredients.

Wound care product	pH of WCP	Active ingredient	Concentration of AI (%) in product	pH of AI
Bactroban	5.9±0.04	Mupirocin	2	3.8±0.02
Colistin milk	3.5±0.02	NT	NA	NA
Flaminal Forte	5.8	NT	NA	NA
Flaminal Hydro	5.8	NT	NA	NA
Flammazine	5.5–5.8	NT	NA	NA
Fucidin	5.2	NT	NA	NA
Furacin	5.9	NT	NA	NA
Hibidil	7.2±0.02	Chlorhexidine gluconate	0.05	6.2
Intrasite Gel	6.4±0.02	NT	NA	NA
Iruxol	7.5	NT	NA	NA
iso-Betadine Gel	2.9	Povidone-iodine	10	2.1±0.02
P.O.H.	4.7±0.02	Oxytetracycline hydrochloridum	0.5	2.8±0.03
		Polymyxin B	0.12	5.8±0.07
Sulfamylon	5.9±0.08	NT	NA	NA

NT, not tested; NA, non-applicable; WCP, wound care product; AI, active ingredient.

Colistin milk contains Polymyxin E as its active component. Polymyxin E is a multicomponent polypeptide antibiotic and comprises of polymyxins A and B. Polymyxins are active against the majority of gram-negative bacteria with some exceptions such as the genera *Neisseria*, *Proteus and Serratia*, which are inherently resistant [20]. Polymyxin E has comparable detergent-like mechanism of action on the outer-membrane of gram-negative bacteria [20–22]. All phages, except myovirus 14–1, got inactivated immediately after getting into contact with colistin milk, with activity dropping below the detection level already after 2 hours, whereas phage 14–1 got inactivated only after 24 hours.

Fucidin contains fusidic acid as its active component, along with hydrocortisone acetate. Fusidic acid is considered as a bacteriostatic antibiotic with relatively narrow antibacterial spectrum, mostly active against gram-positive bacteria and exhibiting good *in vitro* activity against *Mycobacterium tuberculosis* as well [23,24]. Fusidic acid acts as an inhibitor of bacterial protein synthesis by preventing the turnover of elongation factor G (EF-G) from the ribosome [24]. Fucidin barely inactivated phages, whereby the activity of only two of them, i.e. myovirus ISP and podovirus Acibel007, decreased below the ETT titer after 24 hours of incubation (Fig 1, S1 Fig).

P.O.H., also well-known as terra-cortril ointment (Table 2), contains the two antibiotics oxytetracycline hydrochloridum and polymyxin B along with hydrocortisone acetate. Oxytetracycline is a broad-spectrum antibiotic used to treat various kinds of infections. It inhibits translation in bacterial cells by binding to the 30S ribosomal subunit and as such preventing the amino-acyl-tRNA from binding to the A site of the ribosome [25]. As other polymyxins, polymyxin B sulfate acts as a cationic detergent on the bacterial cell wall of gram-negative bacteria, interacting with the lipopolysaccharides of the outer membrane, affecting membrane permeability and causing cell death eventually. Hydrocortisone is a representative of mild corticosteroids and is widely used to treat a broad variety of inflammatory skin disorders [26].

Phages were affected by P.O.H. immediately after incubation, and activity of almost all phages dropped below the DT after 2 hours. The only exception was myovirus 14–1, whose activity decreased gradually and dropped below the DT only after 24 hours (Fig 1, S1 Fig). Two active ingredients of P.O.H., such as oxytetracycline hydrochloridum and polymyxin B were tested separately. Phages were affected differently by these ingredients. Polymyxin B had almost no effect on any of the tested phages, with the exception of ISP, whose activity dropped below the ETT after 24 hours. While oxytetracycline reduced the activity of most of the phages below the ETT immediately or after 2 hours, with the exception of 14–1, which was only inactivated after 4 hours of incubation (S1 Fig). Interestingly, the oxytetracycline solution exhibited an extremely low pH value (2.8) compared to that of the polymyxin B solution (5.8) (Table 3). The differences in pH of between these two active ingredients of P.O.H. could be at the basis of the observed differences in phage inactivation.

Flammazine and Sulfamylon creams contain silver sulfadiazine and mafenide as active ingredients, respectively. Both compounds are representatives of sulfonamides, first widely used synthetic antibiotics. Flammazine cream contains 1% of silver sulfadiazine as a main ingredient and this has broad antimicrobial activity against gram- negative and gram-positive bacteria as well as yeasts. The general mechanism of activity of sulfonamides is based on the inhibition of the bacterial enzyme dihydropteroate synthetase involved in the folate pathway [27]. Silver ions, in turn, are capable to inhibit microbial growth due to several mechanisms such as protein and enzyme inactivation and the blocking of replication and transcription processes by intercalating into DNA molecules [28,29]. However, exact mechanism of activity of silver sulfadiazine has not yet been determined. It is assumed that the overall effect of this compound is due to either the synergetic interaction of sulfadiazine and silver ions or separate activity of each component. Unlike other silver salts, silver sulfadiazine while interacting with sodium chloride containing body fluids (e.g. serum), releases silver ions slowly and sustainably [30], which makes silver sulfadiazine highly effective in the treatment of long-healing wounds, such as burn wounds.

The exact mechanism of mafenide acetate’s activity is also unknown, except that it differs from that of other sulfonamides [27]. Mafenide is particularly effective for the treatment of burn wounds because it is agent that most effectively penetrates the eschar, and is therefore capable of suppressing dense bacterial proliferation beneath the eschar surface. Sulfamylon cream contains 8.5% mafenide acetate, which exerts bacteriostatic action against both gram-negative and gram-positive organisms and which is especially effective against *P*. *aeruginosa* and *Clostridium* spp.

Phage activity was variably affected by Flammazine. *P*. *aeruginosa* phages either were slightly affected or not affected at all. *S*. *aureus* phage ISP appeared to be especially sensitive towards Flammazine and showed gradual decline in activity with a final 4.9 log decrease after 24 hours of incubation. The *A*. *baumannii* phages showed relative tolerance and their titer decreased in the range of 1.3–2.6 log after 24 hours (Fig 1, S1 Fig).

Sulfamylon cream did not much affect phage activity and all phages maintained their activity even after 24 hours of incubation with the exception of the *A*. *baumannii* phages, Acibel004 and Acibel007, for which activity declined by 1.6–1.7 log after 24 hours (Fig 1, S1 Fig).

Another anti-infective topical that contains a synthetic antibiotic is Furacin 0.2%. Nitrofural is the active ingredient of Furacin ointment and, next to being bactericidal it also expresses anti-trypanosomal, anti-fungal activity and it is widely used in human and animal medicine. Nitrofural inhibits several bacterial enzymes, especially those involved in the aerobic and anaerobic degradation of glucose and pyruvate although the exact mechanism of action is unknown [27,31]. Phage activity was not much affected by incubation in Furacin, and only the *A*. *baumannii* phages Acibel004 and Acibel007 showed 2.0 and 0.7 log of decline in activity, respectively after 24 hours of incubation, though the values didn’t drop below the ETT.

Two antiseptics have been tested in our study: Hibidil 0.05% and iso-Betadine Gel 10%. The main component of the antiseptic Hibidil is chlorhexidine, which absorbs onto the microorganism’s cell wall and disrupts the integrity of the cell membrane, causing leakage of intracellular components of the organisms [32]. Hibidil had weaker impact on phage activity than iso-Betadine Gel. The gradual decline in phage activity started after 4 hours of incubation and after 24 hours two phages 14–1 and Acibel004 still maintained activity above the ETT (Fig 1, S1 Fig). The 0.05% chlorehexidine gluconate solution had a similar effect on phages as its final product Hibidil (S1 Fig), even though their pH values differ (7.18 and 6.2, respectively) it must be said that both pH values can be considered to represent a more or less neutral.

Iso-Betadine Gel contains the povidone-iodine complex from which free iodine is slowly liberated, killing eukaryotic and prokaryotic cells through iodination of lipids and oxidation of cytoplasmic and membrane compounds. As a rule, povidone-iodine exhibits a broad range of microbicidal activity against bacteria, fungi, protozoa, and viruses [33]. Iso-Betadine 10% Gel expressed its activity on bacteriophages immediately upon contact and no phage activity could be detected in any samples (even at time zero), except for phage 14–1, which declined with 5.2 log immediately and also dropped below the DT after 2 hours. The 10% povidone-iodine solution also immediately inactivated all phages, with the exception of 14–1, which only dropped below the DT after 2 hours (S1 Fig). Detection threshold values differed slightly between povidone-iodine and its final product iso-Betadine Gel (S1 Fig).

Flaminal Forte, Flaminal Hydro, Intrasite Gel and Iruxol are used for the debridement and desloughing of burn wounds, although the active ingredients of each of them have different modes of activity and in addition Flaminal Forte and Flaminal Hydro also have anti-bacterial properties.

Flaminal Forte and Flaminal Hydro are hydrogels with enhanced wound debridement activity due to the presence of alginate. In addition, the glucose oxidase-lactoperoxidase-guaiacol (GLG) enzyme system confers antimicrobial activity to both products. The difference between Flaminal Forte and Flaminal Hydro is in the concentration of alginate, respectively 5.5 and 3.5%. Phage activity was affected by Flaminal Hydro only after 24 h of incubation but still stayed above the ETT. The decline varied between 1.3–1.9 log for all phages with exception for 14–1, whose activity stayed stable at 9.0 log pfu/ml. The Flaminal Forte formulation had a more pronounced effect on phage activity than Flaminal Hydro. Activity declined far below the ETT after 24 h of incubation and the range of decline varied between 1.7–9.0 log, whereby only myovirus 14–1 maintained activity above the ETT with a final concentration of 8.0 log pfu/ml (Fig 1, S1 Fig).

Intrasite Gel is an amorphous hydrogel which gently rehydrates necrotic tissue of the wound facilitating autolytic debridement. Intrasite Gel is supposed to provide for an optimal moist wound management environment. Intrasite Gel, like Iruxol, had no effect on phage activity.

Iruxol, of which the main component is collagenase, which along with other proteases digests all protein components of the wound thus ensuring chemical debridement, did not affect the activity of any of the tested phages.

We considered the possible correlation between phage activity and pH of the wound care products and a selection of their active ingredients. It is known from previous studies [34] that optimal pH range for most of the representatives of *Caudovirales* active against common bacterial pathogens is 5.0–9.0 [34]. As could be expected, the wound care products and the active ingredients, which exhibited high acidity (below 5.0), i.e. colistin milk, iso-Betadine Gel, P.O.H., mupirocin, oxytetracycline hydrochloride and povidone-iodine (Table 3), had a significant negative impact on phage activity. The most resistant phage appeared to be myovirus 14–1, which belongs to the genus *Pbunavirus*. Representatives of this genus are characterized by acid-resistant capsids resulting in a pronounced resistance to acid environments [12].

Representatives of *Twortvirus* and *Phikmvvirus* genera, to which ISP, PNM and Acibel007 phages respectively belong, are not known to exhibit extraordinary resistance towards pronounced acidic conditions [34–36]. This study confirms these observations.

Precise taxonomic affiliation of myovirus Acibel004 is not defined, most closely related genus is *Kpp10virus* [10]. However, there is not much data available on biophysical stability of representatives of this genus, which makes comparison difficult. Based on our study Acibel004 proved to be quite sensitive to low pH values too, though it is not the most sensitive one among the five tested phages. Phages ISP and Acibel007 exhibit the least stability in most of the experiments conducted during the study.

## Conclusions

In conclusion, each of the burn wound care products expressed variable activity on the tested phages. No correlation was detected between morphology, host range of phages and their stability towards the wound care products. The myovirus 14–1, active against *P*. *aeruginosa* strains, appeared to be the most stable phage, of which the activity in most of the assays stayed above the ETT during at least 4 hours of incubation, with iso-Betadine Gel as the only exception. The other four phages expressed variable but more or less similar tolerance towards all tested products. Phages were mostly hampered by anti-infective products, such as Bactroban, colistin milk, iso-Betadine and P.O.H. ointment. It should be noted that all wound care products have a complex composition, and that—besides the active components—they contain a variable number of different additives, conferring stabilizing effects to the active components. This makes it difficult to speculate which particular components of the tested products are responsible for phage inactivation. Certain combinations of various ingredients might also have inactivation effects. The results also suggest a correlation between the acidity of certain burn wound products and phage inactivation. Before applying phage preparation along with common burn wound care products careful stability/activity analysis should be carried out as every phage expresses different levels of tolerance/resistance towards different products. Simultaneous application of some products, especially of those with antiseptic activity and high acidity, along with phages should be avoided. However phage preparations can still be applied with most of the products and will stay active during at least 4 hours of incubation. Phages can absolutely be recommended to be applied with neutral hydrogels aimed for wound debridement and not-containing any anti-infective agents. Nevertheless, the tests performed in the scope of this study provide information only about *in vitro* activity and further *in vivo* experiments or clinical trials are needed to prove efficacy of joint application of phage and conventional therapy products in treatment of burn wounds.

## Supporting information

S1 Fig Stability of bacteriophages in burn wound care products and a selection of their active ingredients (in alphabetical order). **A) Burn wound care products; B) Active ingredients.** The results are the mean values of three titrations. Standard deviations are indicated. Detection thresholds for each product and each bacterial species are indicated by color gradient columns. ETT: effective therapeutic titer (7.0 log pfu/ml).
(PDF)
