# Supplementary figures and images for: Stability of bacteriophages in burn wound care products
Source: PLoS One. 2017 Jul 27;12(7):e0182121. doi: 10.1371/journal.pone.0182121 (PMC5531522; doi:10.1371/journal.pone.0182121)

## A) Wound care products

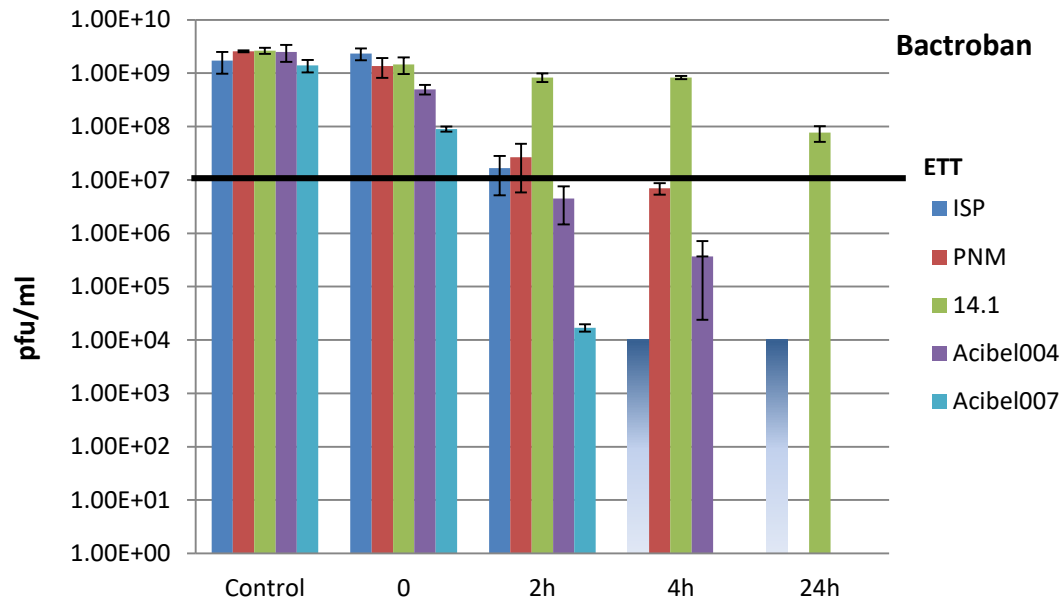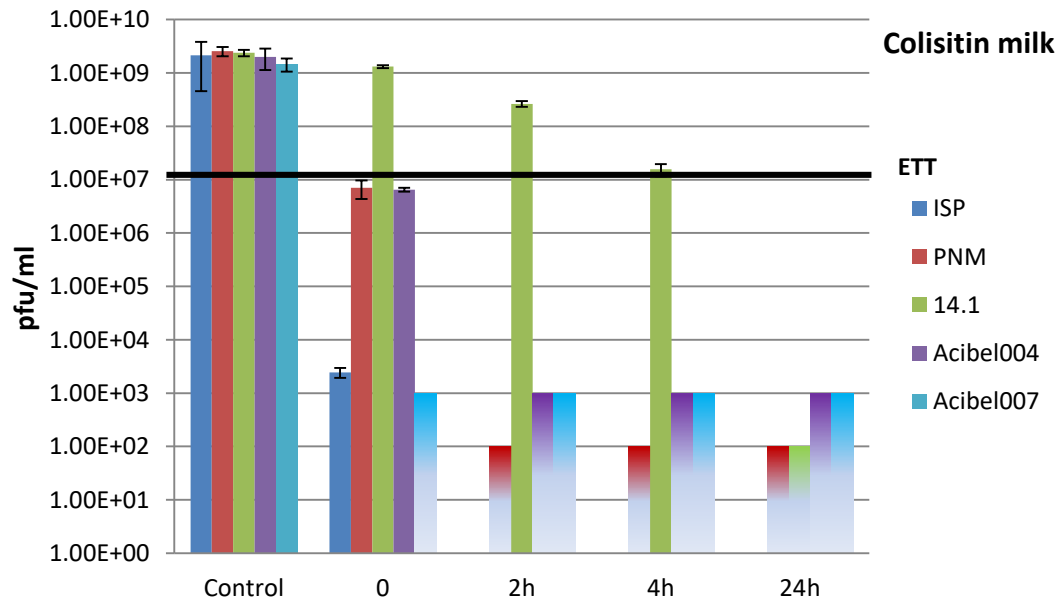

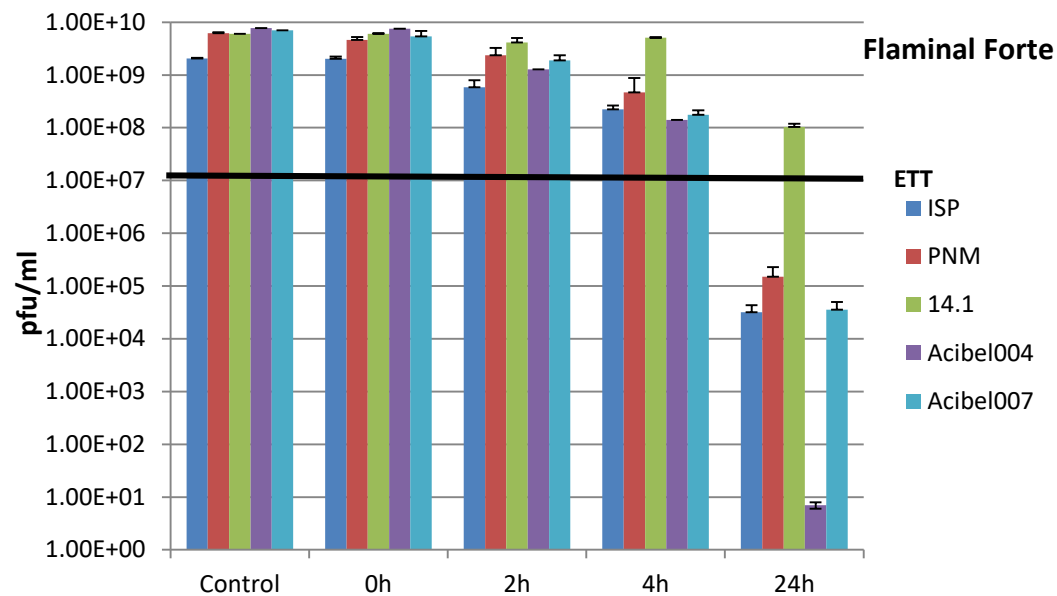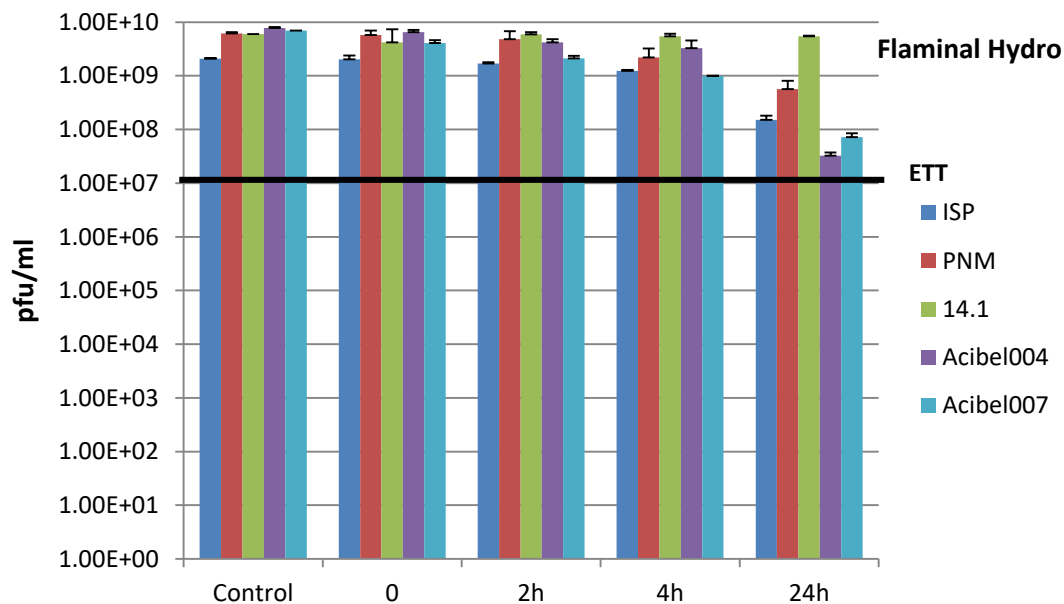

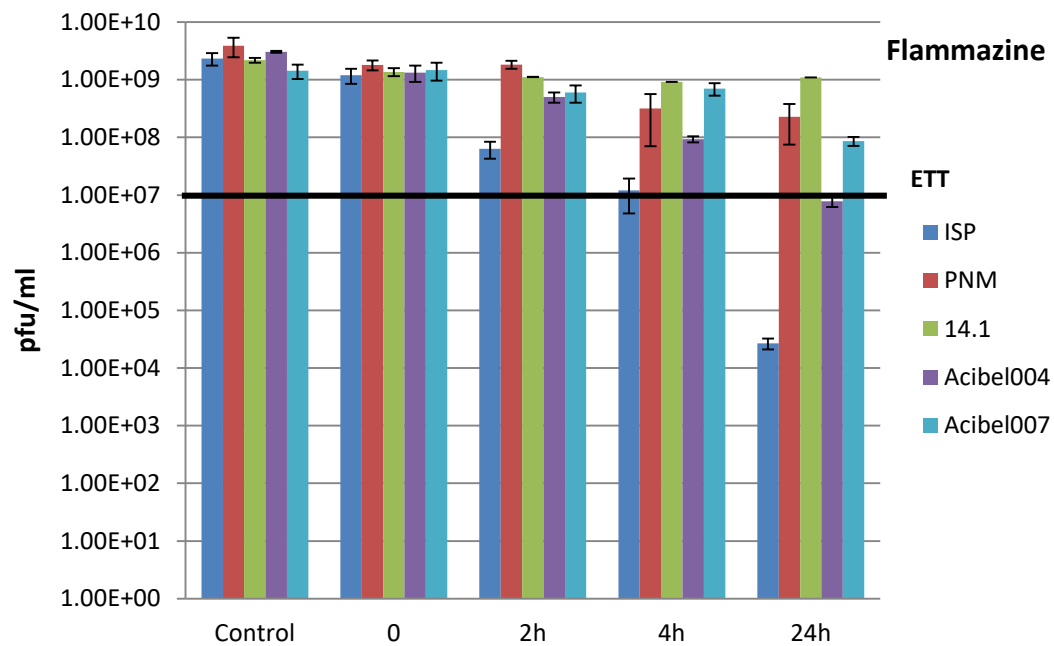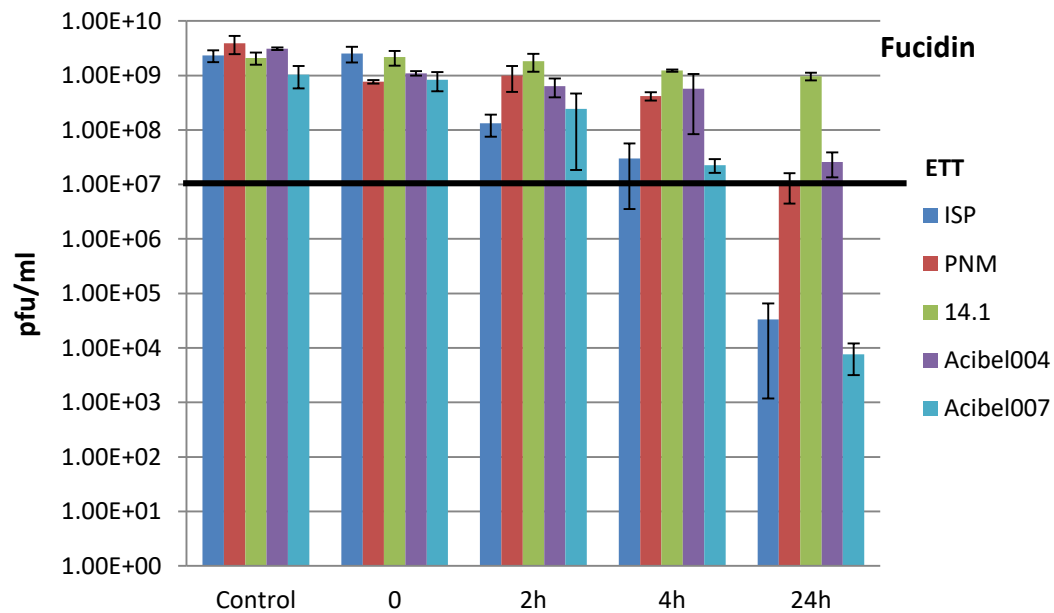

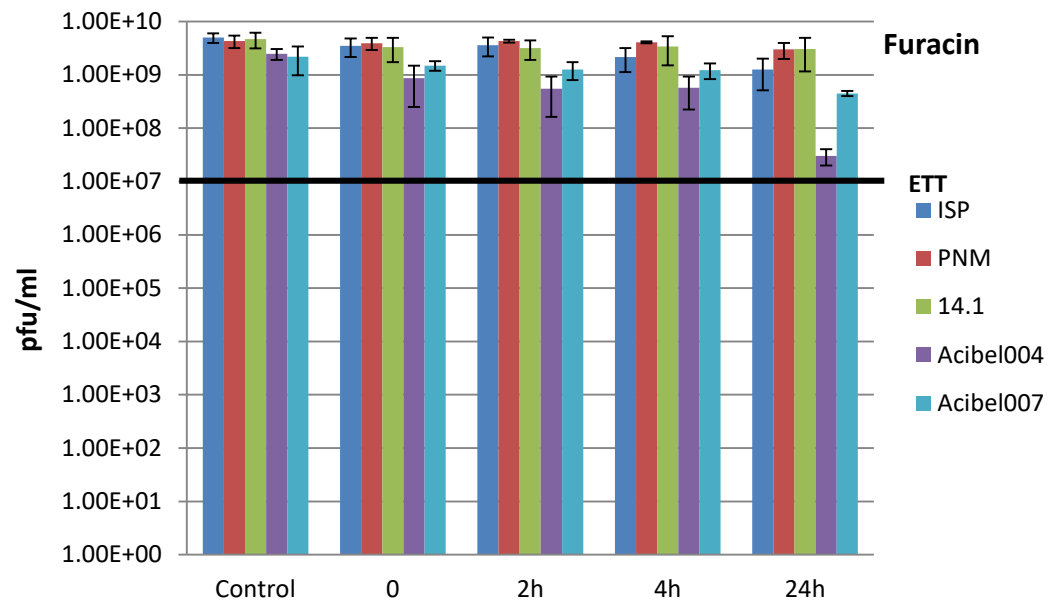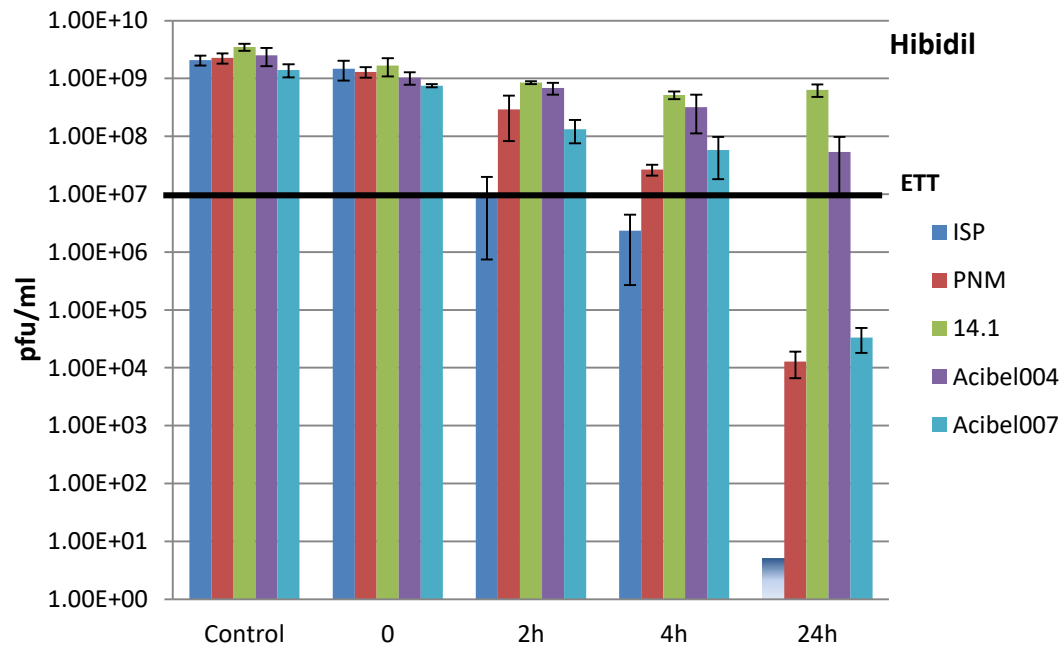

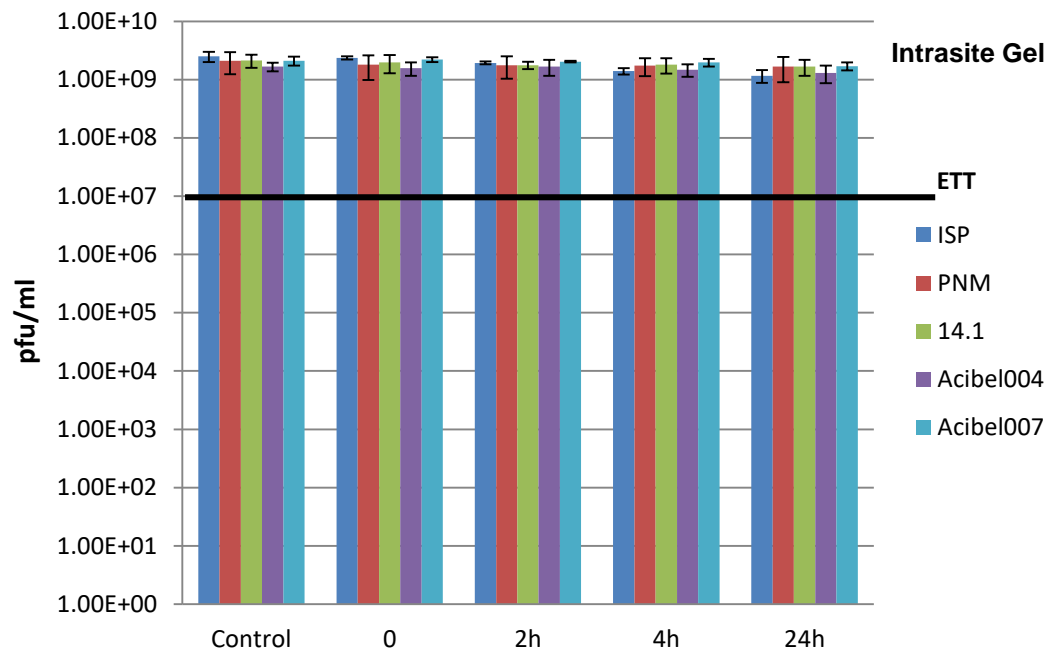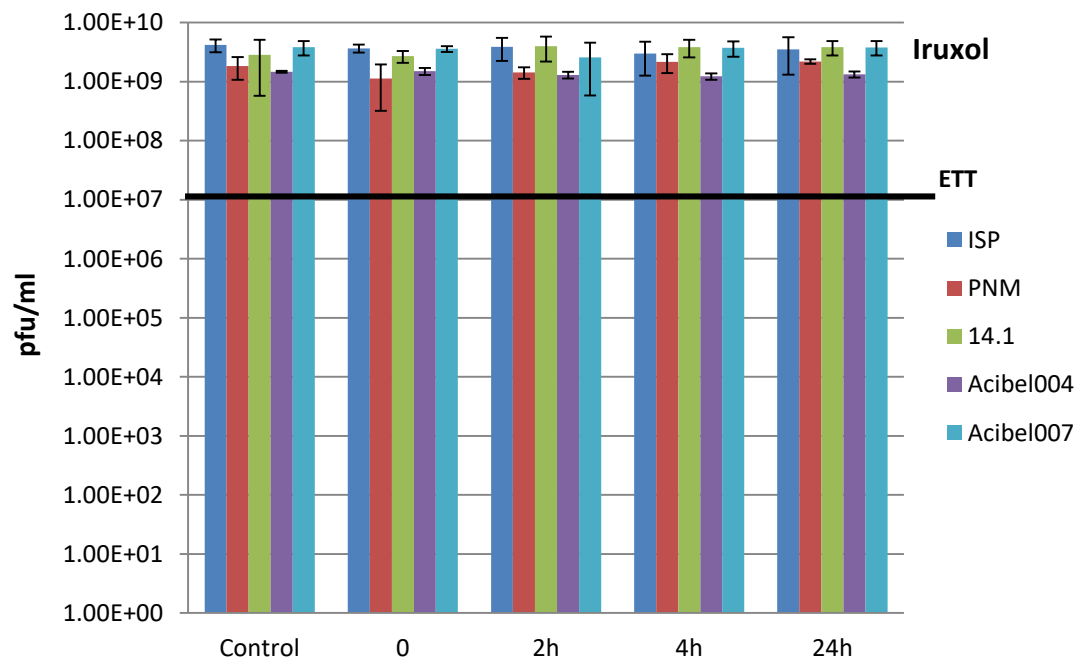

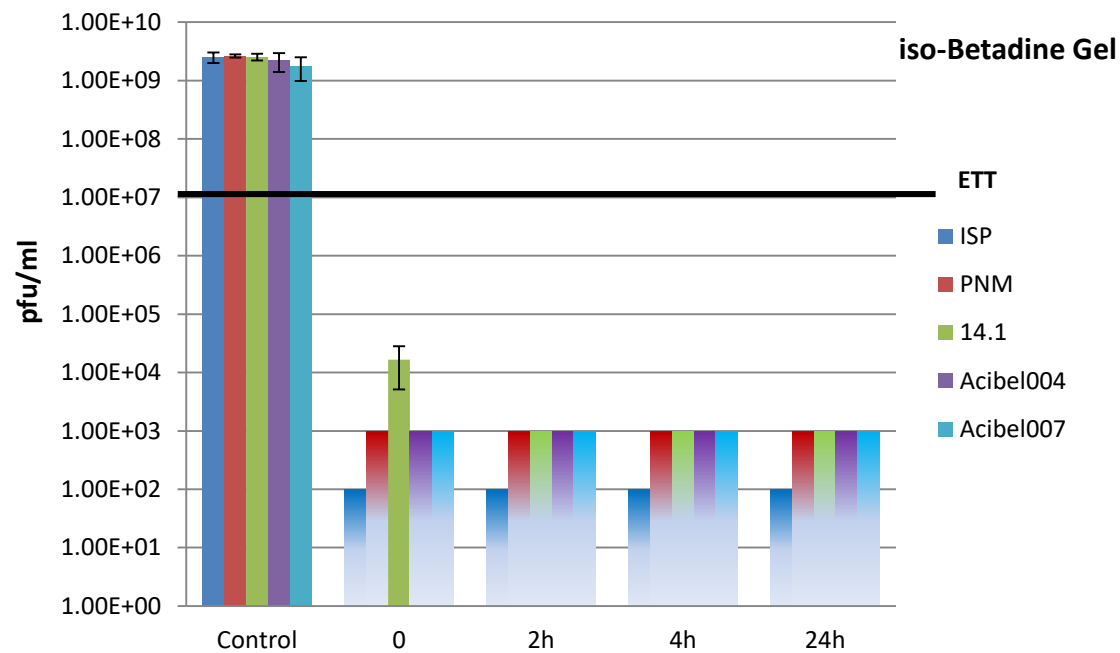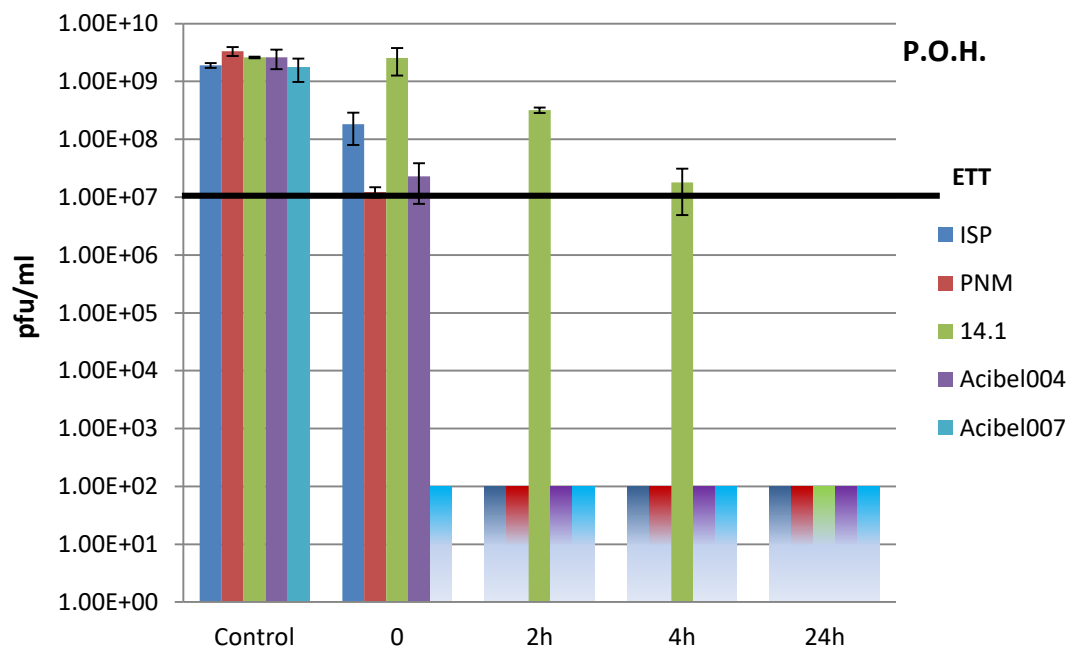

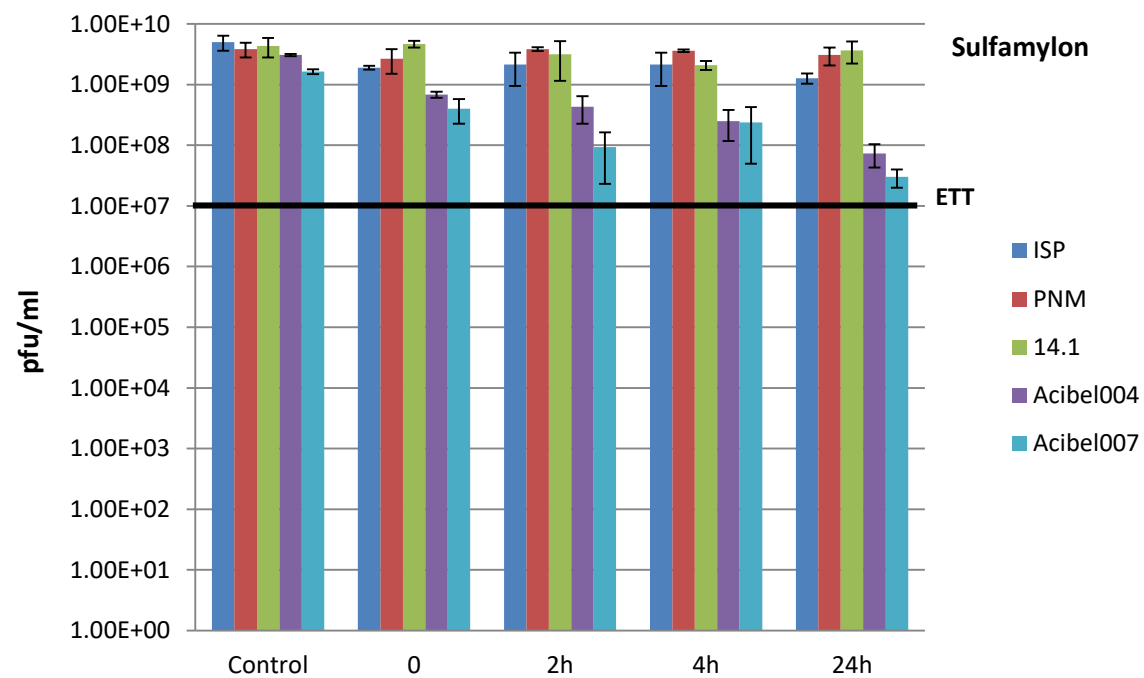

## B) Active ingredients

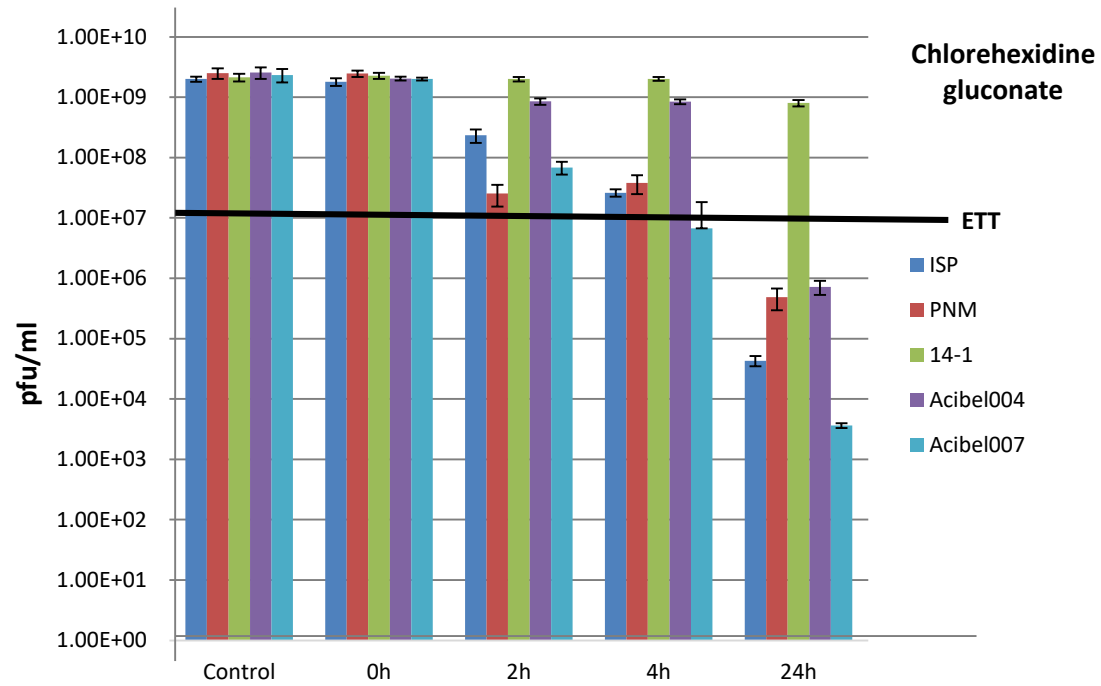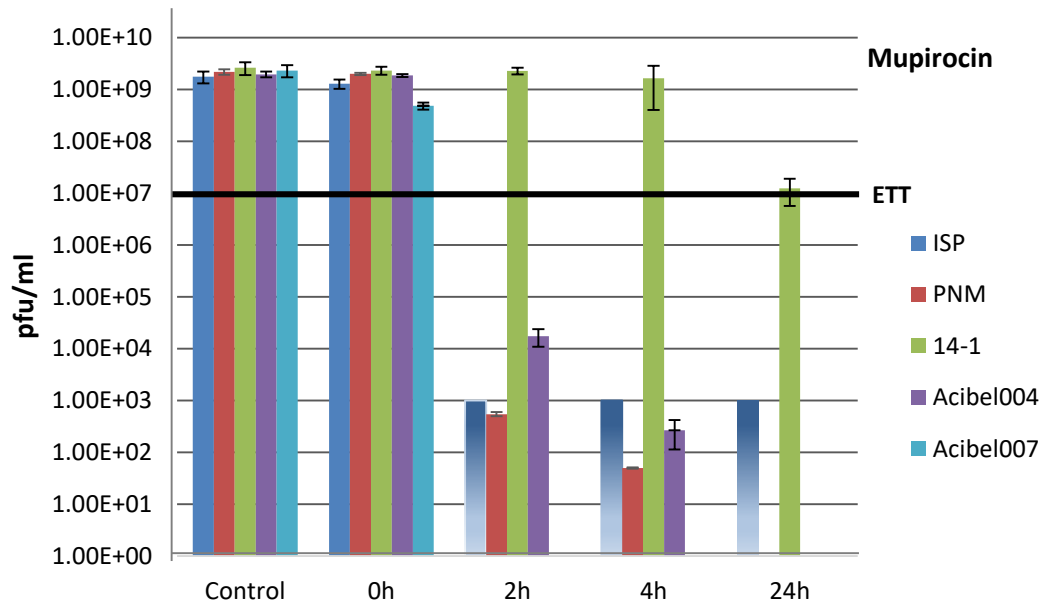

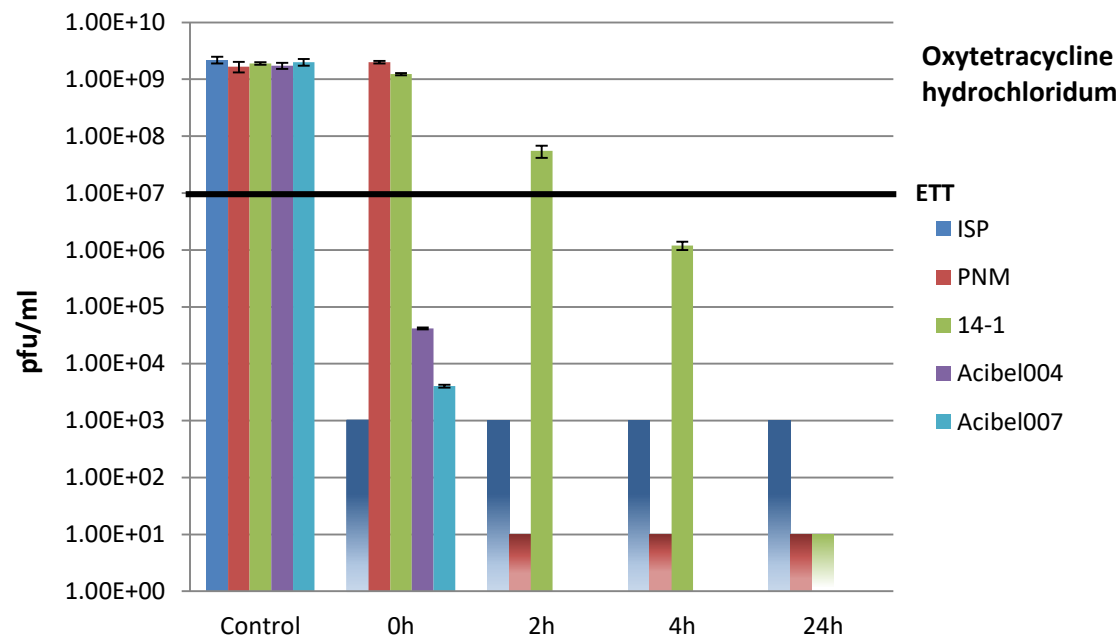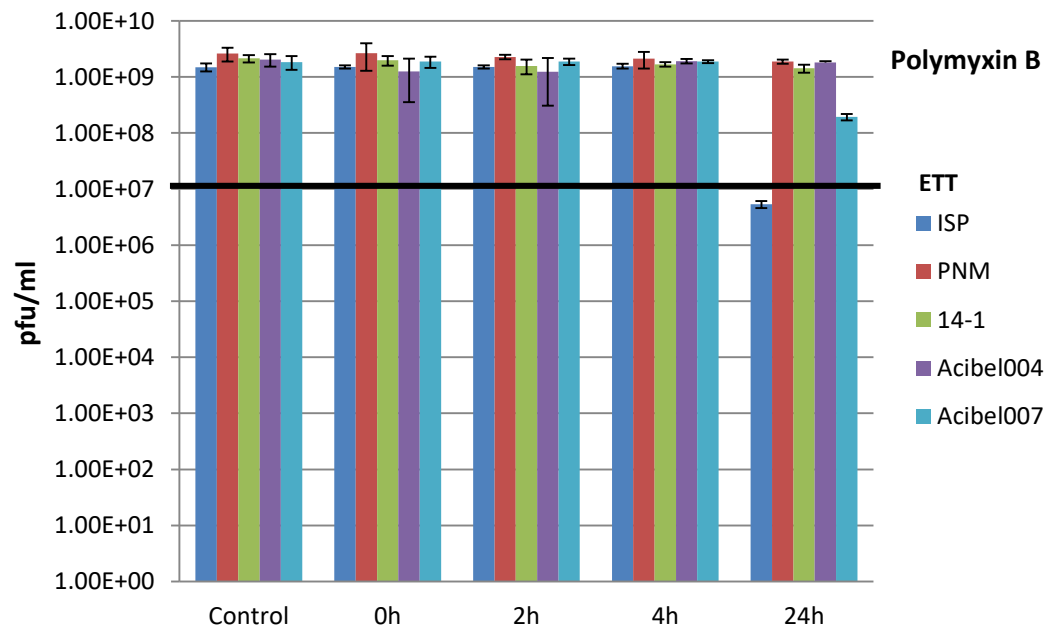

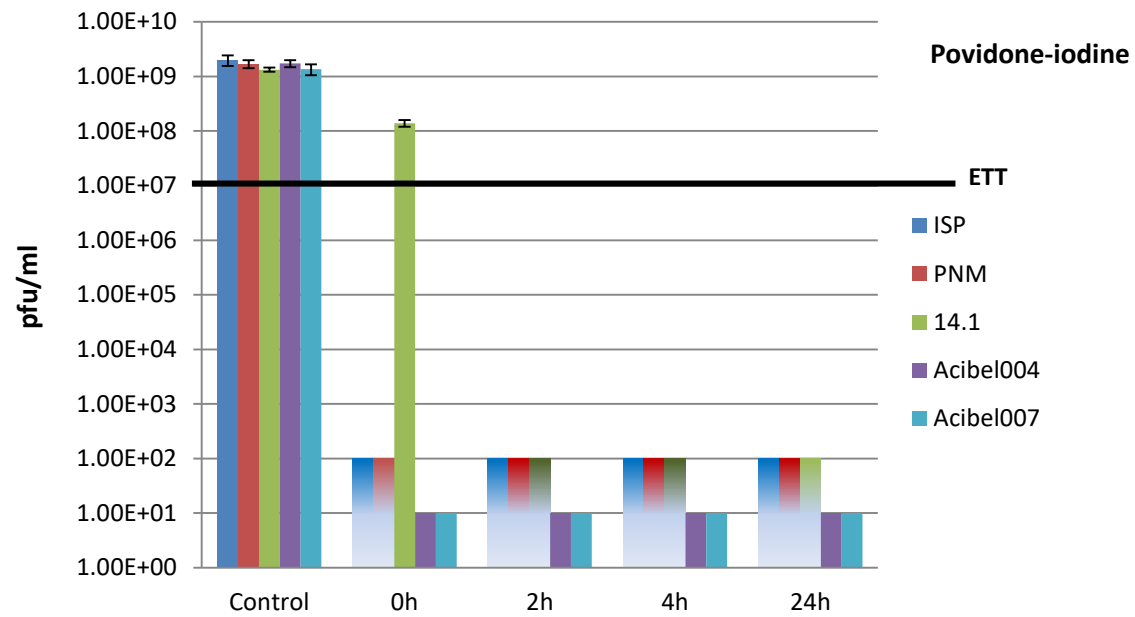

Supplement: S1 Fig — A) Burn wound care products; B) Active ingredients. The results are the mean values of three titrations. Standard deviations are indicated. Detection thresholds for each product and each bacterial species are indicated by color gradient columns. ETT: effective therapeutic titer (7.0 log pfu/ml). (PDF) [file pone.0182121.s001.pdf]
